# “I prefer to take pills when I plan to have sex”: Perceptions of on-demand versus daily oral pre-exposure prophylaxis among adolescents in Kampala, Uganda

**DOI:** 10.2989/16085906.2022.2039727

**Published:** 2022-03-01

**Authors:** Rachel Kawuma, Zam Nabalwanyi, Janet Seeley, Yunia Mayanja

**Affiliations:** 1 Medical Research Council/Uganda Virus Research Institute and London School of Hygiene and Tropical Medicine Uganda Research Unit, Entebbe, Uganda; 2 London School of Hygiene and Tropical Medicine, London, United Kingdom

**Keywords:** acceptability, adolescents, high risk, HIV prevention, PrEP regimen and preferences, young people

## Abstract

There is limited information about the use of on-demand and daily pre-exposure prophylaxis (PrEP) among adolescents and young people (AYP) in sub-Saharan Africa. We explored perceptions of both regimens among 14- to 19-year-olds perceived to be at high risk of HIV infection in Kampala, Uganda, using qualitative data collection methods. Data were analysed by theme and interpreted based on constructs from the framework of acceptability. Although there were no noticeable gender differences in preferences for a particular regimen, acceptability of PrEP depended on individual AYP sexual behaviour at the time of the study. Those who perceived themselves to be at increased risk of acquiring HIV preferred using daily PrEP, citing the consistency that comes from taking a pill daily and which they considered to be efficacious and safe. AYP who had less frequent sex preferred on-demand PrEP because it would enable them to “plan for sex”. However, both groups perceived taking daily PrEP to be a burden, which was an impediment to acceptance of this form of PrEP. AYP anticipated that daily pill taking would be very stressful, requiring a lot of effort and would interrupt their daily routine. Therefore, while both on-demand and daily PrEP were acceptable and beneficial to these AYP, preferences for either regimen depended on self-perceived risk. Thus, oral PrEP use should be tailored to end-user preferences and risk profiles.

## Introduction

Despite a global reduction in new HIV infections over the past decade, annual infections in sub-Saharan Africa (SSA) remain high at 870 000, with adolescents and young people (AYP) accounting for 25% of these new infections (UNAIDS, 2020). Studies among AYP in SSA show that a high proportion report early sexual debut (before 15 years old) which exposes them to HIV, sexually transmitted infections (STIs) and pregnancy (Mmbaga et al., 2012; Idele et al., 2014; Yakubu & Salisu, 2018). In Uganda, more males than females (15% compared to 10.4%) were likely to report early sexual debut before the age of 15 (Seff et al., 2021).

Early sexual debut is associated with factors that increase risk for HIV infection among females when compared to males, such as condom-less sex, multiple and age-disparate sexual relationships, intimate partner violence and transactional sex. These factors heighten underlying biological risks due to an immature cervix and genital trauma from STIs and forced sex (Pettifor et al., 2009; Stöckl et al., 2013; Winston et al., 2015).

In 2020, among 15- to 19-year-olds in eastern and southern Africa, only 27% of females and 16% of males were tested for HIV and had received their last test result in the past 12 months (UNICEF, 2020). With this low prevalence of HIV testing and inconsistent condom use reported in eastern Africa (Idele et al., 2014), the introduction of novel biomedical HIV prevention interventions such as oral pre-exposure prophylaxis (PrEP) are timely. Indeed, in 2018, the World Health Organisation (WHO) developed specific guidelines for provision of daily oral PrEP to AYP, particularly adolescent girls and young women, in settings with a high burden of HIV infections.

Early reports from PrEP implementation projects among adolescent girls and young women in SSA showed that oral PrEP uptake was lower than expected (Dunbar et al., 2018), and did not achieve the high uptake levels seen in clinical trials (Celum et al., 2018). Community-based studies of daily oral PrEP implementation in East Africa also indicate lower uptake among people younger than 25 years old (Mugwanya et al., 2019; Koss et al., 2020). In addition, recent findings from the Combined HIV Adolescent PrEP and Prevention study (CHAPS) conducted in Uganda, Zimbabwe and South Africa indicate that even though young people were willing to use PrEP, there are barriers at individual, interpersonal, community, institutional and structural levels that require multi-level interventions to promote PrEP use (Muhumuza et al., 2021).

Haberer et al. (2015) suggest that to achieve prevention-effective use, PrEP use must be understood in the context of use of other HIV prevention methods and dynamic HIV risk profiles. This is because individual HIV risks change over time and daily PrEP use during times of low risk unnecessarily exposes individuals to costs, side effects and toxicity while reducing availability to others (Haberer et al., 2015; Molina et al., 2015). Adoption of non-daily PrEP regimens, also referred to as on-demand or event-driven, may be more effective and efficient for some individuals as shown in an open label study among adult men who have sex with men (MSM) in the United States (Molina et al., 2015). Conversely, a randomised trial done among at-risk adult heterosexual women in which they were assigned to different self-administered oral PrEP regimens showed that drug adherence, coverage of sex acts and drug concentrations in blood were higher with daily PrEP regimens (Bekker et al., 2018).

Non-daily regimens have the advantage of fewer side effects and reduced pill burden (Molina et al., 2015; Bekker et al., 2018). However, these regimens are not widely studied among adolescents in SSA and therefore data are still limited. To contribute to filling this gap, we conducted a study using qualitative data collection methods that assessed perceptions of on-demand and daily oral PrEP among HIV-negative 14–19-year-old adolescents perceived to be at high risk of HIV infection enrolled in a cohort study in Kampala, Uganda.

### Theoretical framework

**Figure 1:**
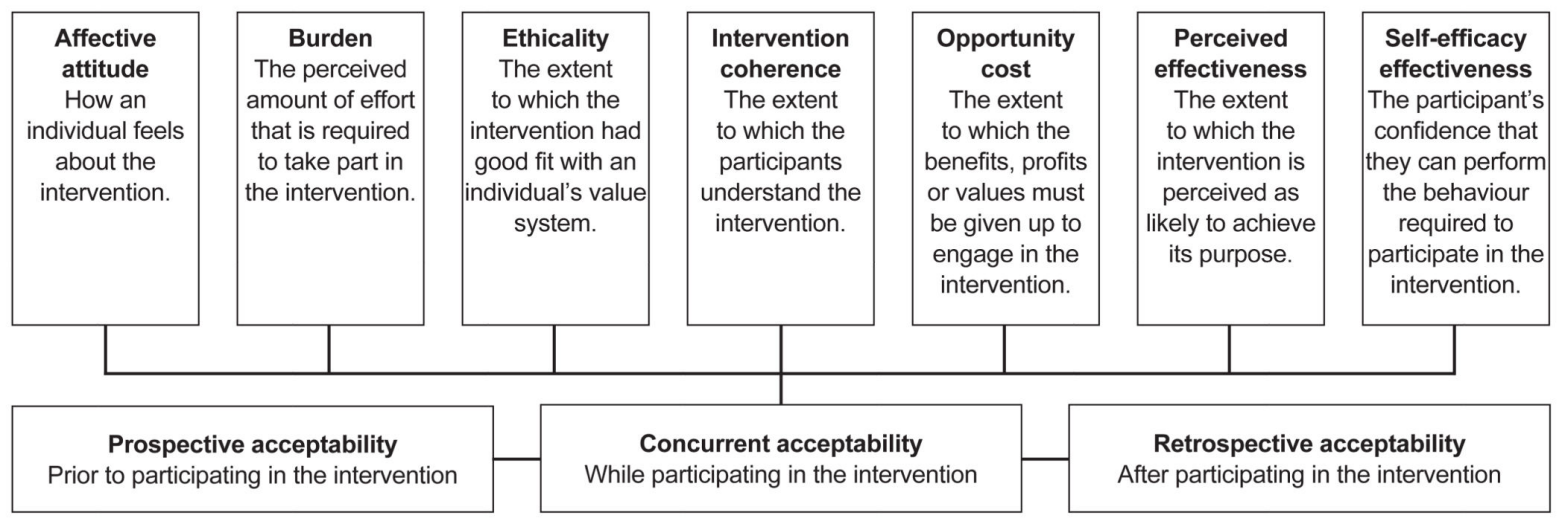
The theoretical framework of acceptability (Adapted from Sekhon et al., 2017)

We used the theoretical framework of acceptability (TFA) to assess participant preference for on demand or daily PrEP regimen. The TFA (Figure 1) explores the extent “to which people delivering or receiving a health intervention consider it to be appropriate based on anticipated or experienced cognitive and emotional responses to that intervention” (Sekhon et al., p. 3). This framework has been used elsewhere to assess attitudes towards interventions and uptake of services (Murphy & Gardner, 2019; Pavlova et al., 2020).

## Methods

### Study setting

We gathered views from young people who were part of the **"F**easibility of **e**nrolling and **r**etaining a**d**olescents **a**t **r**isk” (FERDAR) study about their preferences of either on-demand or daily PrEP. FERDAR is an adolescent cohort study aimed at assessing the feasibility of enrolling and retaining 500 (200 males and 300 females) 14–19-year-old adolescents at risk of HIV infection. The study assessed hypothetical willingness to use either daily or on-demand PrEP. However, because daily oral PrEP (tenofovir/ lamivudine) is the approved regimen in Uganda and was available at the clinic, young people who opted to start were offered daily oral PrEP throughout the duration of the study. As part of the study procedures, health workers provided information to participants, highlighting the difference between daily and on-demand oral PrEP before any data collection.

The cohort was based at the Good Health for Women Project (GHWP) clinic (Vandepitte et al., 2011; Mbonye et al., 2013) in southern Kampala, Uganda, from March 2019 to December 2020. Young people were identified and recruited from sex work locations, bars and lodges and densely populated areas of poor housing where alcohol and drug misuse were common. Characteristics used to assess eligibility for the study included emancipated or mature minors of under 18 years old as defined in national research guidance (Uganda National Council for Science and Technology, 2014), sexually active in the past three months and willing to return to the clinic for follow-up visits.

### Sampling and recruitment

Ten per cent (30 females and 20 males) of the adolescents in the FERDAR study were purposively selected to participate in 50 in-depth interviews (IDIs). In addition, 52 (27 females and 25 males) who did not take part in IDIs participated in six focus group discussions (FGDs; 3 males and 3 females). Recruitment was conducted during the clinic visits where social science researchers working closely with study nurses identified participants for both IDIs and FGDs. They included participants of different genders, ages, work types and from different locations in Kampala.

### Data collection

Both FGDs and IDIs were used to gather information in this study. Similar key areas of investigation were covered using both methods. These included knowledge about PrEP, preferences for either regimen or assessment of risk perception.

All interviews were conducted in private by three experienced social scientists (two females and one male) all younger than 30 years old and proficient in Luganda, the main local language. The FGDs lasted 60 to 90 minutes, while the IDIs lasted 40 to 60 minutes. They were all audio-recorded, transcribed and translated verbatim and transcripts were cross-checked against the audio recordings to ensure accuracy.

### Data management and analysis

Following the principles of thematic analysis (Braun & Clarke, 2006), emerging themes in the data were identified and coded manually into an Excel sheet code book to identify recurring patterns. Two members of the study initially read four transcripts to familiarise themselves with the contents and identify codes which were discussed by the entire team during a debriefing meeting. Codes with similar or close meanings were grouped together to generate broader themes, after which a coding matrix was developed. Thereafter, the rest of the scripts were read and excerpts charted on a matrix under relevant or matching themes to enable quick and easy navigation. Four dominant themes emerged during analysis and these included “Frequency of sex", “Nature of relationship/s", “Locus of control", and “Nature of the pill". The themes were then charted in relation to the constructs of TFA which we found matched with five constructs of the framework, namely intervention coherence, affective attitude, self-efficacy, perceived effectiveness and burden. The other two constructs of ethicality and opportunity costs did not emerge from the data as themes, so we have excluded them from our adapted version of the TFA. We present the overall findings and the excerpts to support and illustrate the findings identified with either IDI or FGD of a female or male.

### Ethics statement

The Uganda Virus Research Institute Research Ethics Committee (GC/127/18/09/658) and the Uganda National Council of Science and Technology (HS 2493) approved the study. All participants provided written informed consent with those unable to read or write indicating consent with a thumb print in the presence of a witness independent of the study. All minors 14 to 17 years old were included if identified as emancipated or mature (according to national guidelines, as noted above) and able to provide independent consent. Participants were informed about their rights to withdraw from the study at any time and that this would not affect any health care that they were entitled to.

## Results

### Participant’s characteristics

As shown in Table 1, the majority of the 30 (18F, 12M) participants who took part in the qualitative component of the FERDAR study were 18 and 19 years old. All had dropped out of school at the time of this study with half (26) having attained primary level education (13F, 13M), while 20 (14F, 6M) had secondary level education. Four did not have any formal education (3F, 1M). Many described their occupation as self-employed (37; 19F, 18M) with the rest saying they were not working. The females worked as waitresses in bars and others were street food vendors. Males, on the other hand, mainly did manual unskilled work, e.g. casual labourers at construction sites or plastic and metal scrap collectors. Only 11 females said they were in regular relationships, often describing themselves as “married", while the rest and all the males were not “married". Five females mentioned that they started using daily oral PrEP after joining the study. Overall, PrEP was acceptable among these young people and preference for a particular regimen was interpreted in relation to their current sexual behaviour. The findings are presented below for each TFA of the five constructs which corresponded to our data.

### Affective attitude

This construct relates to how study participants felt about using either on-demand or daily PrEP. Relating each PrEP regimen to their frequency of having sex, they preferred one that would be convenient for their current lifestyle. For instance, when asked about their preferred regimen, both males and females commonly responded depending on how regularly they had sex: *“I prefer daily PrEP to on-demand PrEP because I stay with my boyfriend and we often have sex”* (FGD, females, 14–15 years old) and “*I prefer on-demand PrEP because I rarely have sex. I can go a month without having sex”.* (IDI, male, 19 years old).

Secondly, having the option of either on-demand or daily PrEP comes with flexibility as it allows one to be able to “plan” for when one would be able to use PrEP as expressed in the following quote: *“I would choose on-demand PrEP because you take it when you plan to have sex, not on a daily basis. Daily PrEP puts you under pressure; taking it daily is tiresome, whereas on-demand, you take when you plan to have sex”* (FGD, males, 18-19 years old).

### Intervention coherence

Described as the extent to which people recognise the aim of the intervention and how it works, all participants acknowledged that using PrEP, whether on-demand or daily, would benefit them because they would be able to protect themselves from getting HIV. They reflected on the nature of the relationship(s) they were in, whether regular or irregular, while stating their preferences for a regimen.

For instance, some young people felt that daily PrEP was most suited for people with regular partners who are likely to have sex more frequently, such as couples in stable relationships, “*I prefer daily PrEP because I am married and I anticipate regular sex. Anytime my husband comes, I am ready to have sex”* (IDI, female, 17 years old) and those engaged in sex work, “*I prefer daily PrEP because of the nature of my job, I am a sex worker and I usually get daily customers, so daily PrEP is best for me”* (IDI, female, 19 years old).

However, those who did not live with or regularly have sex with their partners preferred on-demand PrEP, “*I prefer on-demand PrEP because I have inconsistent partners”* (FGD, males, 14–15 years old). “*I prefer on-demand to daily PrEP because my boyfriend comes once in a while; I can't take daily PrEP because my boyfriend is inconsistent”* (FGD, females, 14–15 years old). One of the young women summarises this view thus:

On-demand, you have to take it when you don't stay with your husband and it is not appropriate for married people. Because for married people, you can't be without having sex. On-demand is appropriate for people who don't stay with their partner; it is appropriate when you just pay him or her a visit. You take it when you are going to visit him and you plan to have sex with him but when you are staying with your husband, it is better you take daily PrEP (FGD, female, 18–19 years old).

### Perceived effectiveness

Perceived effectiveness is the extent to which the intervention is perceived as likely to achieve its goal. Having understood that the aim of PrEP was to protect them from getting HIV, some females actually started using daily oral PrEP, particularly because they noted the risk that comes with the nature of the sex work that they were engaged in. They also considered having consistent sexual partners which would require a more consistent method of prevention as opposed to the on-demand regimen which they viewed as inconvenient because one had to time sex before taking it. Other than the protection it offers, they also pointed to the consistency that comes with taking a pill which they considered to be efficacious and safe. “*I prefer daily PrEP because I am still in the business (sex work) and I regularly have sexual intercourse”* (IDI, female, 19 years old). Another emphasised that:

I would prefer to take daily PrEP because taking it [daily PrEP] is routine; you take it daily and it will be hard for me to forget. For daily PrEP you can move with it and it is not hard to take as long as you choose a convenient time to take it. But for on-demand PrEP, you may forget the actual time you took the pills because you have to count 24 hours from the time you took the pills (IDI, female, 19 years old).

### Self-efficacy

This is a TFA construct defined as the participant's confidence that they can perform the behaviour required to participate in the intervention. In this study, having control over decisions regarding when and with whom one has sex was an important aspect that influenced preference for a PrEP regimen. Being able to “plan” for when and with whom to have sex was a main thread that ran through most of the interviews. Those with irregular partners preferred on-demand PrEP because they only had to take it when they would have sex “I *prefer on-demand PrEP because I don't have a regular* [partner] *man. I take it when I plan to have sex or get a man to have sex with”* (IDI, female, 18 years old). On the other hand, those who did not seem to have control over when they would have sex preferred to take PrEP daily. For instance, one of the young women said *“I prefer daily PrEP because you never know when you are going to have sex. He may come abruptly when you have not yet taken on-demand PrEP”* (IDI, female, 17 years old).

Daily PrEP was also preferred by the males, having recognised that there were periods when they could not use condoms either because of cost or being high on drugs. Below are two excerpts from FGDs with male participants (16–17 years old):

Daily PrEP is good and it is best for boys who have daily sex. When you take daily PrEP, you don't have to stress yourself buying condoms. Sometimes we lack money to buy condoms; you end up having “live” sex. But when you take pills on time, there is no need to look for money to buy condoms. You will be safe and you will have “sweet” sex without minding the condom.

I take drugs [marijuana] and when I take drugs, I fail to control myself, hence having unprotected sex with girls or women I find [that come my way]. So that means when I have unprotected sex with several girls or women, when I have already taken it [daily PrEP], it will give me protection and I will not worry about getting infected.

### Burden

Both the perceived and experienced burden of taking PrEP was expressed by participants. The perceived burden arose from assessment of the information provided by the study staff about the two PrEP regimens. Many participants were concerned about the pill burden associated with a daily regimen; they anticipated that this would be very stressful, would require a lot of effort to take and was uncomfortable because it would likely interrupt their daily routine. One of the young men said:

We like oral PrEP but taking daily pills is a challenge. I prefer on-demand because I don't want to take unnecessary drugs [pills] when I am not going to have sex (FGD, male, 18–19 years old).

In addition, they were concerned about the reaction of family members if they saw them taking PrEP which could be perceived as anti-retroviral (ARV) drugs since they look similar. In this case, they preferred taking on-demand PrEP which did not require being taken frequently and which they could try to take without others noticing.

For me I prefer on-demand to daily PrEP because my parents are very strict. The PrEP tin is like that of ARVs and they will be wondering, why I am taking daily pills. They will think that I got HIV. It is better for me to take on-demand PrEP (FGD, females, 18–19 years old).

On the other hand, those who had either taken PrEP themselves or had heard or witnessed challenges encountered by others taking it explained the burden they had felt or had seen others experience. To them, associating the pill with the ones taken by people who are HIV positive was the main concern. One of the females who started taking oral PrEP in the study mentioned this:

The packaging scares me; I hide the tin whenever I come for PrEP, this is because I don't want people to know that I take PrEP. They will think that I am on ARVs, yet it is PrEP. I hide the tin and when you are with me, you can't tell that I have it in the bag. When I pick up the PrEP, I come alone when there is no participant at the clinic so that no one knows that I have come to pick up PrEP. I wake up very early in the morning so that I make it to the clinic when it's still early and no one has arrived. By the time they [participants] come I have already finished and I am going back home (IDI, female, 19 years old).

In addition, another young woman (14–15 years old) commented during an FGD:

My friend went before me to the clinic and when she came back home, she told me about PrEP and I asked her to show me the pill because I wanted to see what it looks like. When she showed me the pill, I got scared because it size is so big and is like ARVs. The size of the pill discouraged me to ask for daily [ordinary] PrEP.

Therefore, we note that PrEP was generally acceptable in principle, but the reality of taking PrEP, for those who had experience, showed that the burden associated with daily PrEP will likely continue to be a major impediment to uptake and adherence.

## Discussion

We found that PrEP is generally acceptable to young people, as has been noted elsewhere (Camlin et al., 2020; Maseko et al., 2020) because the AYP we spoke to said that they desired a method that would protect them against HIV. Preference for using either the on-demand or daily regimen depended on one's sexual behaviour at the time of the study and also the experience of having taken PrEP or having known people who had taken PrEP. It has been argued that oral PrEP acceptability and adherence should be tailored to the sexual behaviour of an individual as this allows them the ability to improve their self-efficacy (Farrington et al., 2016; Cornelisse et al., 2019). Many young people considered the frequency with which they had sex as the benchmark for a regimen, arguing that the more one had sex, the more they are exposed to risk of acquiring HIV. Indeed, many epidemiological studies (Farrington et al., 2016; Giovenco et al., 2018) have considered the frequency of sex while assessing risk.

Awareness of risk is known to increase PrEP acceptability (Maseko et al., 2020). In our study, some of the young females involved in sex work started taking daily PrEP provided in the study because they were concerned about risk. Similarly, some males pointed to situations of risk where they had had sex without using condoms and said they preferred daily PrEP. This preference was primarily because PrEP provides them with a sense of being “safe” at all times since they were sometimes unable to predict when sex would occur (Reyniers et al., 2018), and the consistency that comes with taking a pill which they considered safe and efficacious.

While we generally did not find any gendered preference for either PrEP regimen, there were underlying stereotypes often showing a lack of autonomy when it comes to making sexual decisions by females, as documented elsewhere (Duby et al., 2021). On the other hand, for the males, concerns about upholding their masculinity related to reputation and respectability influenced their PrEP preferences (Siu et al., 2013; Mbonye et al., 2021) such as a preference for condom-less sex which would be facilitated by taking PrEP to protect themselves from HIV infection.

Due to the spontaneity of sex in some situations, it may sometimes be difficult to “plan", thereby affecting decisions regarding PrEP uptake as documented with other interventions like family planning (Hoggart & Phillips, 2011). Secondly, the on-demand regimen would require one to take several pills during pre-specified hours before sex. This regimen becomes complex to follow and may affect the regimen efficacy. Therefore people who opt for on-demand PrEP need support to stick to it and to maximise efficacy, as noted by Giovenco et al. (2018).

One major challenge to acceptability of PrEP in this study, as has been noted elsewhere (Kawuma et al., 2021; Muhumuza et al., 2021), was taking pills which are similar to those taken by people living with HIV. Hence participants preferred on-demand PrEP, largely because it would shelter them from being seen “taking pills daily". Relatedly, Tanner et al. (2020, p. 2) observe that “efficacy of PrEP is a function of adherence” and is influenced by the stage of adolescence a young person has reached.

The AYP also preferred the “flexibility” and “convenience” associated with either PrEP regimen and were keen on one that would suit their risk profile at a given time. For instance, in previous studies of PrEP regimen preferences, participants acknowledged that they would switch their regimen in future depending on their risk profile (Reyniers et al., 2018; Cornelisse et al., 2019). Risk is not static and adolescents in our study were able to state their preference for a PrEP regimen based on their perceived HIV risk and on their experience of its use if they had used PrEP before.

Using a modified version of TFA allowed us to draw out key themes around acceptability of either PrEP regimen. However, our data did not provide material to discuss two constructs of TFA. We did not include “ethicality” because the AYP did not talk about their value systems. Nor did “opportunity cost” resonate with our findings because most participants were discussing hypothetical situations and they were also all part of a study with benefits and therefore did not incur any opportunity costs.

### Strengths and limitations

The strength of this study was the ability to get young people thought to be at risk of acquiring HIV to discuss their preference for a novel HIV prevention method. Despite many of them being PrEP naive, a few started using daily oral PrEP provided at the clinic, while others knew friends who were using it. These experiences informed their discussions. The main limitation was that only a few had actual experience of using PrEP and since only the daily PrEP regimen was approved, they did not have a chance to experience on-demand PrEP use. Secondly, they discussed preference based on current behaviour rather than on the long-term benefits that PrEP could afford them.

## Conclusion

Adolescents at risk of HIV infection perceive oral PrEP use to be acceptable and beneficial to them. Preferences for on-demand or daily regimens depend on their perceptions of risk at a given time. Therefore, tailoring oral PrEP use to end-user risk profiles will likely increase uptake and adherence.

## Figures and Tables

**Table 1:** Characteristics of study participants

Participant characteristics	Number (*n*)
Gender	
Females (F)	30
Males (M)	20
Age	
16–17 years old	20 (12F, 8M)
18–19 years old	30 (18F, 12M)
Education	
No formal education	4 (3F, 1M)
Primary education	26 (13F, 13M)
Secondary education	20 (14F, 6M)
Occupation	
Self-employed	37 (19F, 18M)
No work	13 (11F, 2M)
Relationship type	
Regular “married	11 (only females)
“Not married”	39 (19F, 20M)
